# Pantothenate and CoA biosynthesis in Apicomplexa and their promise as antiparasitic drug targets

**DOI:** 10.1371/journal.ppat.1010124

**Published:** 2021-12-30

**Authors:** Laura E. de Vries, Matteo Lunghi, Aarti Krishnan, Taco W. A. Kooij, Dominique Soldati-Favre

**Affiliations:** 1 Department of Medical Microbiology, Radboudumc Center for Infectious Diseases, Radboud Institute for Molecular Life Sciences, Radboud University Medical Center, Nijmegen, the Netherlands; 2 Department of Microbiology & Molecular Medicine, Faculty of Medicine, University of Geneva, Geneva, Switzerland; University of Georgia, UNITED STATES

## Abstract

The Apicomplexa phylum comprises thousands of distinct intracellular parasite species, including coccidians, haemosporidians, piroplasms, and cryptosporidia. These parasites are characterized by complex and divergent life cycles occupying a variety of host niches. Consequently, they exhibit distinct adaptations to the differences in nutritional availabilities, either relying on biosynthetic pathways or by salvaging metabolites from their host. Pantothenate (Pan, vitamin B5) is the precursor for the synthesis of an essential cofactor, coenzyme A (CoA), but among the apicomplexans, only the coccidian subgroup has the ability to synthesize Pan. While the pathway to synthesize CoA from Pan is largely conserved across all branches of life, there are differences in the redundancy of enzymes and possible alternative pathways to generate CoA from Pan. Impeding the scavenge of Pan and synthesis of Pan and CoA have been long recognized as potential targets for antimicrobial drug development, but in order to fully exploit these critical pathways, it is important to understand such differences. Recently, a potent class of pantothenamides (PanAms), Pan analogs, which target CoA-utilizing enzymes, has entered antimalarial preclinical development. The potential of PanAms to target multiple downstream pathways make them a promising compound class as broad antiparasitic drugs against other apicomplexans. In this review, we summarize the recent advances in understanding the Pan and CoA biosynthesis pathways, and the suitability of these pathways as drug targets in Apicomplexa, with a particular focus on the cyst-forming coccidian, *Toxoplasma gondii*, and the haemosporidian, *Plasmodium falciparum*.

## Author summary

The Apicomplexa phylum comprises thousands of parasitic species, among which the causative agents of malaria, toxoplasmosis and cryptosporidiosis. New parasiticidal compounds and drugs are urgently needed for treatment of these devastating diseases. As these parasites have adapted innovative pathways for nutrient acquisition, several studies have investigated vitamins and cofactor synthesis and salvage, with the aim of identifying unexplored drug targets. Coenzyme A (CoA) is an essential cofactor for cell biology and is synthesized from pantothenate (Pan, vitamin B5). The discovery of the druggability of CoA synthesis in *Plasmodium falciparum* has sparked intensive research toward lead compounds identification and preclinical development. Here, we review the current literature on the topic from biological and pharmacological perspectives. Focusing on *Plasmodium* species and *Toxoplasma gondii*, we describe recent findings on the importance of Pan synthesis, salvage, and metabolization to CoA in this phylum. In addition, we summarize recent promising advances in the exploration and exploitation of these pathways for lead compounds optimization and drug development.

## Introduction

The Apicomplexa phylum encompasses a large and diverse group of parasites exhibiting distinct lifestyles within one or more cellular niches and hosts. In humans, these parasites can cause debilitating and deadly diseases such as malaria, toxoplasmosis, and cryptosporidiosis [1–3]. Arguably, the most successful zoonotic parasite is *Toxoplasma gondii*, with the highly proliferative form (tachyzoites) capable of infecting virtually all warm-blooded animals and replicating in most nucleated cell types. Upon encountering immune pressure from the intermediate host, the tachyzoites are rapidly eradicated, while few of them differentiate into the slow growing, cyst-forming bradyzoites that are responsible for chronic infection to ensure persistence and transmission [4,5]. Malaria is caused by parasites of the genus *Plasmodium*, which reside mainly intracellularly in the vertebrate host, while extracellularly in the mosquito host. First, the malaria parasite develops in liver cells in humans, and after release, parasites multiply via a continuous cycle of asexual replication in red blood cells. Some of these parasites enter a different developmental program and form sexual stages (gametocytes) that are required for successful colonization of a mosquito, the site of sexual reproduction [6,7]. Intracellular *Plasmodium* and *Toxoplasma* parasites have several ways to scavenge nutrients from the host, through the induction of new permeation pathways (NPPs) in *Plasmodium*-infected erythrocytes, the selectively permeable parasitophorous vacuole membrane, and by membrane transporter proteins [8–14]. Understanding the acquisition and *de novo* synthesis of metabolites and the characterization of metabolic pathways for the production of essential vitamins or cofactors is of significant therapeutic interest [15]. In this review, we focus on recent advances in our understanding of the biology of the pathways for the synthesis of pantothenate (Pan, vitamin B5) and coenzyme A (CoA) in the apicomplexan parasites *T*. *gondii* and *P*. *falciparum*, and their exploration as antiparasitic drug targets.

### Pantothenate biosynthesis occurs only in the coccidian branch of the Apicomplexa

**Fig 1:**
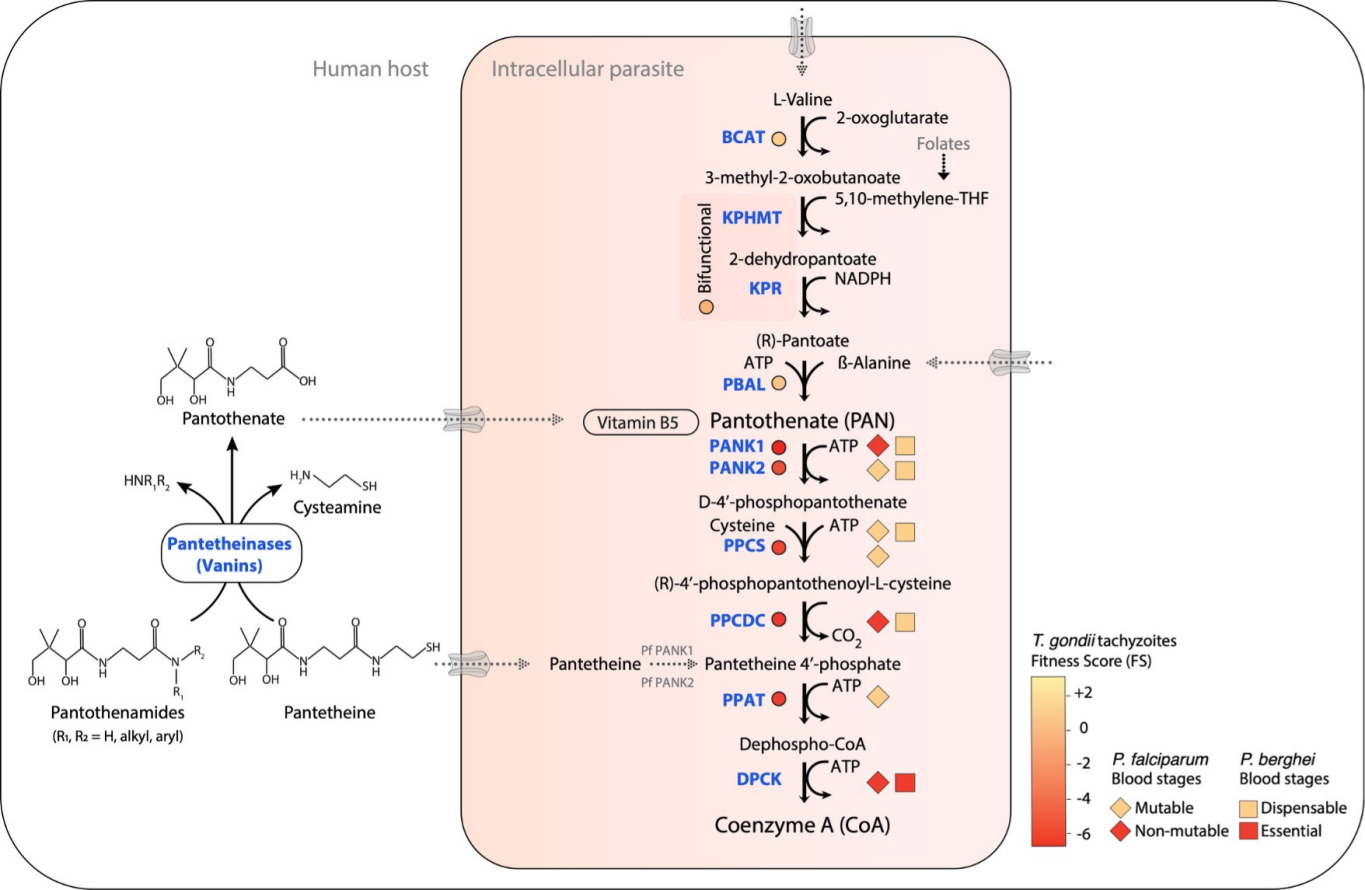
Pan and CoA biosynthesis pathways and the predicted essentiality in *T*. *gondii*, *P*. *berghei*, and *P*. *falciparum*. Phenotypic data for *T*. *gondii* tachyzoites and *P*. *berghei* and *P*. *falciparum* asexual blood stages are derived from Sidik and colleagues [20], Bushell and colleagues [21], and Zhang and colleagues [22], respectively. Since the essentiality data for each of the species (*T*. *gondii*, *P*. *berghei*, and *P*. *falciparum*) were obtained using different genetic approaches, the most representative forms are displayed. The FS for *T*. *gondii* (circles) is an experimentally observed measure (ranging from −6.9 to +3 for the fitness cost associated with the disruption of a given gene for parasite survival. Fitness-conferring genes display a lower FS and dispensable genes display a higher FS [20]. Phenotypic data for *P*. *falciparum* genes based on their MIS, which estimates the potential mutability of a gene, are represented as diamonds [22]. For *P*. *berghei*, a gene is indicated as dispensable or essential based on the relative growth rate observed upon genetic disruption within the asexual blood stage (squares) [21]. Dashed lines represent putative routes. Pantothenamides and pantetheine can further be hydrolyzed into Pan via the action of host-encoded pantetheinases (vanins) [23]. BCAT, branched-chain amino acid transaminase; CoA, coenzyme A; DPCK, dephospho-CoA kinase; FS, fitness score; KPHMT, ketopantoate hydroxymethyltransferase; KPR, α-ketopantoate reductase; MIS, mutagenesis index score; PBAL, pantoate-β-alanine ligase; Pan, pantothenate; PANK, pantothenate kinase; PPAT, phosphopantetheine adenylyltransferase; PPCDC, phosphopantothenoylcysteine decarboxylase; PPCS, phosphopantothenoylcysteine synthetase.

**Fig 2:**
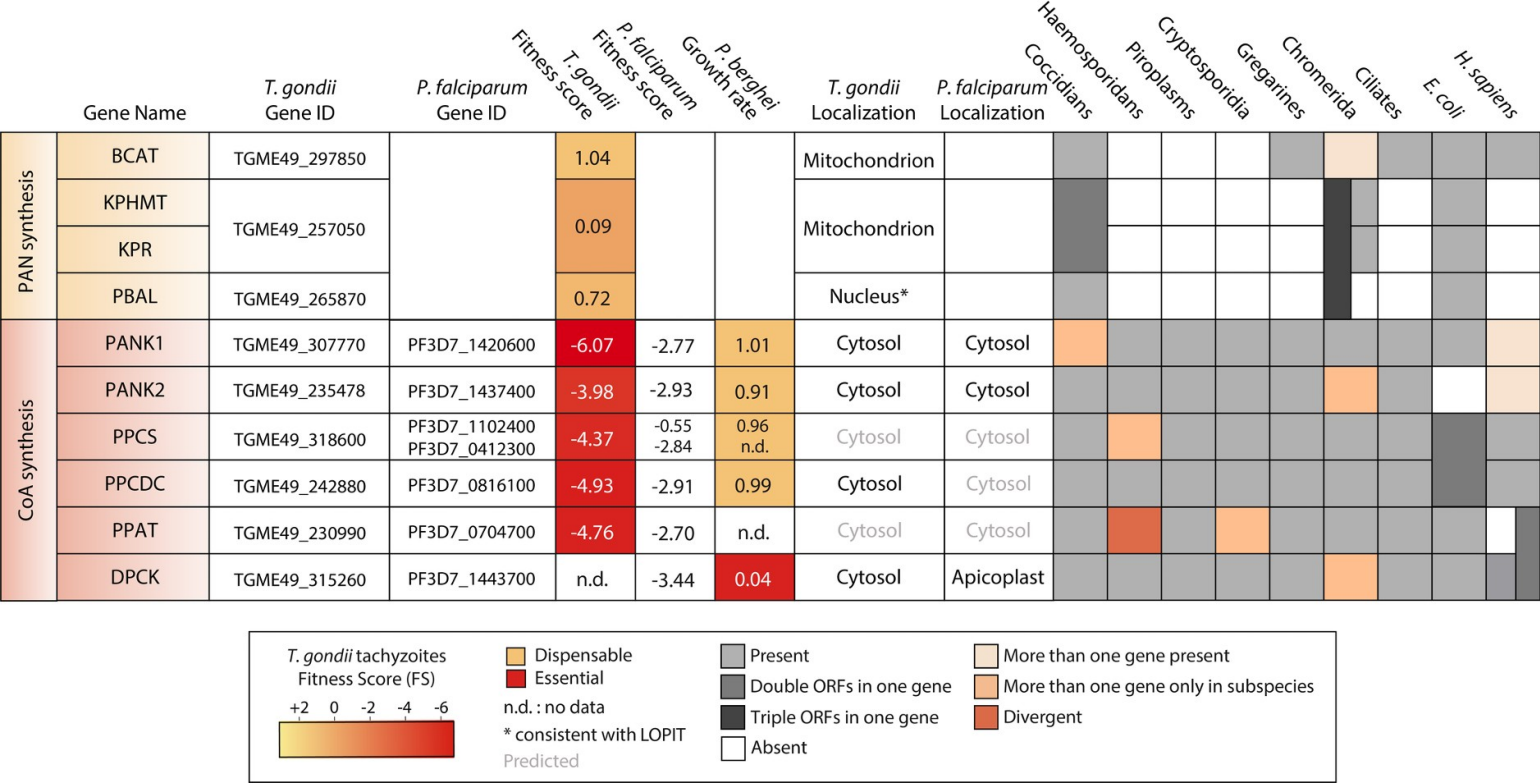
Genes encoding Pan and CoA biosynthesis enzymes. Phenotypic data of *T*. *gondii* tachyzoites and *P*. *berghei* and *P*. *falciparum* asexual blood stages are derived from Sidik and colleagues [20], Bushell and colleagues [21], and Zhang and colleagues [22], respectively. Since the essentiality data for each of the species (*T*. *gondii*, *P*. *falciparum*, and *P*. *berghei*) were obtained using different genetic approaches, the scores are not comparable. The FS for *T*. *gondii* is an experimentally observed measure (ranging from −6.9 to +3) for the fitness cost associated with the disruption of a given gene for parasite survival. Fitness-conferring genes display a low FS and dispensable genes display a high FS [20]. The presented fitness score for *P*. *falciparum* is based on the MFS, which estimates the relative growth fitness cost for mutating a gene based on its normalized quantitative insertion-site sequencing reads distribution. The scores range from −4.1 to 2.8, with lower scores indicating nonmutability of a gene [22]. Experimental localization data (in black) were obtained from Oppenheim and colleagues [34], Lunghi and colleagues [16], and Barylyuk and colleagues (only PBAL is identified in this study) [17] for *T*. *gondii*, Tjhin and colleagues [35] and Swift and colleagues [36] for *P*. *falciparum*. Predicted localization is shown in gray. Variability in conservation is shown with different colors: light gray when only 1 copy of the gene is present, gray when 2 ORFs are present in a single gene, and dark gray when 3 ORFs exist in a single gene. Light orange is when more than 1 gene is identified in the whole group for the indicated catalytic activity, orange when only some species within the group encode more than 1 gene, and dark orange when the gene can be identified but has poor sequence similarity to the genes found in other species. BCAT, branched-chain amino acid transaminase; CoA, coenzyme A; DPCK, dephospho-CoA kinase; FS, fitness score; KPHMT, ketopantoate hydroxymethyltransferase; KPR, α-ketopantoate reductase; LOPIT, localization of organelle proteins by isotope tagging; MFS, mutagenesis fitness score; Pan, pantothenate; PANK, pantothenate kinase; PBAL, pantoate-β-alanine ligase; PPAT, phosphopantetheine adenylyltransferase; PPCDC, phosphopantothenoylcysteine decarboxylase; PPCS, phosphopantothenoylcysteine synthetase.

Pan is the precursor for the biosynthesis of the essential cofactor CoA. Most bacteria, plants and fungi, can synthesize it *de novo*, while animals need to acquire this vitamin from their diet. Pan is synthesized from the branched-chain amino acid, L-valine, which undergoes 3 consecutive biotransformation reactions: (i) a hydroxymethyl transfer of ketoisovalerate, a metabolite of valine, to form α-ketopantoate; (ii) reduction of α-ketopantoate to pantoate; and (iii) ligation of pantoate with β-alanine (commonly derived from L-aspartate) to form Pan (Fig 1). These reactions are performed by the enzymes ketopantoate hydroxymethyl transferase (KPHMT), ketopantoate reductase (KPR), and pantoate-β-alanine ligase (PBAL, also called Pan synthetase), respectively. Whereas other apicomplexans lack this pathway altogether, the coccidians, including *T*. *gondii*, encode the enzymes catalyzing the 3 Pan synthesis reactions in only 2 genes, with the first 2 transformations of the pathway catalyzed by a bifunctional KPHMT-KPR enzyme (Fig 2). In non-apicomplexan alveolates, Pan synthesis genes are duplicated and organized in several ORFs. The photosynthetic symbionts *Vitrella brassicaformis* and *Chromera velia* contain *KPHMT* and *KPR* as single genes, as well as in 1 trifunctional ORF also comprising *PBAL*. The *KPHMT-KPR* fusion can also be observed in the oyster parasite *Perkinsus marinus*, in which PBAL activity is encoded by 2 genes instead [15]. Recent works in *T*. *gondii* on the localization of KPHMT-KPR and PBAL revealed that these enzymes localize to the mitochondrion and nucleus, respectively [16,17]. The branched-chain-aminotransferase (BCAT), which generates α-ketoisovalerate from L-valine, also localizes to the mitochondrion [18], possibly pointing toward the initiation of Pan synthesis within this organelle. The localization of PBAL in the nucleus is surprising but is confirmed by a proteome-wide fractionation study (localization of organelle proteins by isotope tagging (LOPIT) [17]), which reports localization for only this enzyme in the pathway. However, the precursors and product of the enzyme are expected to diffuse freely between the cytosol and nucleus. Previously, a pharmacological study described the Pan synthesis pathway in *T*. *gondii* and proposed its druggability by testing the efficacy of compounds designed to target *Mycobacterium tuberculosis* Pan synthetase [19]. Consistent with whole-genome fitness score study performed via CRISPR-Cas9 editing in *T*. *gondii* [20], and in sharp contrast to the pharmacological evidence [19], *KPHMT-KPR* and *PBAL* were found to be fully dispensable in *in vitro* cultured tachyzoites, as well as in the mouse model of acute infection [16]. Moreover, while PBAL has been shown to be a functional enzyme *in vitro* and *in vivo*, stable isotope labeling experiments demonstrated that Pan synthesis is not occurring in *in vitro* standard cultured *T*. *gondii* tachyzoites and that parasites are dependent on Pan salvage from the host cell. Remarkably, given the dispensability and inactivity of the synthesis pathway in tachyzoites, individual deletion of either *KPHMT-KPR* or *PBAL* gene in the cyst-forming type II (ME49) strain of *T*. *gondii* resulted in the dramatic reduction in the number of cysts in the mouse brain, clearly supporting the utilization of Pan synthesis in bradyzoites [16].

The retention of the Pan synthesis pathway exclusively in coccidians and the critical role of the Pan synthesis pathway for establishment of chronic infection by *T*. *gondii* are intriguing. Perhaps, the preferential niches occupied by bradyzoites have limited Pan levels, leaving scarce amounts for scavenge by the parasite. The most studied route of synthesis of β-alanine in bacteria is from aspartate by aspartate decarboxylase [24] and in yeast by degradation of spermine [25]. In mammalian organisms, β-alanine can be synthesized by a variety of routes, such as a 3-enzymatic step from uracil [26], from purine catabolism via the β-alanine synthetase [27], and from glutamate by glutamic acid decarboxylase–like 1 enzyme [28]. While β-alanine accumulates in brain tissues [29] and plays a role as neurotransmitter [30], it is also necessary for synthesis of the histidine-containing dipeptides carnosine and anserine [31], both of which have physiological roles as an antioxidant, pH buffer, and neurotransmitter, and accumulate in brain and muscle tissues as well [32]. Mining of the *Toxoplasma* genome failed to identify homologous enzymes capable of synthesizing β-alanine via the various routes known in mammalian, bacterial, or yeast systems. Concordantly, *T*. *gondii* tachyzoites are dependent on exogenous β-alanine to produce Pan and PBAL activity could only be identified upon supplementation with both pantoate and β-alanine [16]. Furthermore, uptake of labeled β-alanine and formation of Pan was only observed in parasites expressing PBAL [16]. The presence of a highly divergent pathway, active in a different life cycle stage of the parasite where β-alanine supply is limited, cannot be ruled out. However, given that *Toxoplasma* cysts reside in β-alanine and carnosine-rich tissues [30], it is plausible that a selective pressure has led to the retention of an active Pan synthesis and acquisition of its precursors from the host. Pan is a small metabolite with a MW of 219 Da that presumably diffuses freely through the molecular sieve of the parasitophorous vacuole membrane [9,33]. Accessibility of host-derived nutrients to encysted bradyzoites is currently unknown, and reduced permeability across the cyst wall and limited diffusion through large cysts could explain the necessity for Pan biosynthesis. The precise transport mechanism and molecular entity mediating Pan transport to the intracellular environment, as well as the molecular basis for *Toxoplasma* tissue preference for cyst formation, remain unanswered.

### In search of a pantothenate transporter in Apicomplexa

All apicomplexans are expected to rely on salvage of Pan from the host. This has been demonstrated in *T*. *gondii* by mass isotope labeling experiments [16], and a seminal study also clearly demonstrated that *P*. *falciparum* requires exogenous Pan for growth [37]. Pan transport is increased drastically in infected compared to uninfected red blood cells and is mediated by the NPP [38]. The existence of a Na^+^-independent, pH-dependent Pan transporter in *P*. *falciparum* has been formally demonstrated [39], and, therefore, inhibiting the Pan transporter presents a promising drug-based intervention strategy. However, the transporter remains unidentified. In model organisms, highly divergent Pan transporters have been described, including the Na^+^ /Pan symporter PanF in *Escherichia coli* [40], a major facilitator superfamily transporter Fen2 in yeast [41] and a Na^+^-dependent multivitamin transporter SLC5A6 in mammals [42]. Surprisingly, PanF, Fen2, and SLC5A6 share no sequence similarity, and, therefore, it is plausible that apicomplexans harbor yet another divergent Pan transporter. An attempt to identify the *P*. *falciparum* Pan transporter by homology with Fen2 led to the characterization of a putative transporter, *Pf*PAT, although with a relatively poor homology [43]. While the scavenging of exogenous Pan is essential for *P*. *falciparum* growth in erythrocytes, PAT is dispensable for blood-stage growth of rodent malaria parasites and only essential for mosquito transmission [44,45]. Later studies revealed a major role of *Pb*PAT (and its homolog TFP1 in *T*. *gondii*) in secretion of osmiophilic bodies and microneme maturation, respectively [46,47], rendering its role as a Pan transporter highly unlikely. A complementary strategy to the homology search for the identification of the Pan transporter could take advantage of the currently available genome-wide datasets, the LOPIT dataset for *T*. *gondii* [17] and large-scale genetic screens [20–22]. The candidate list would be limited to candidates with the following: (i) plasma membrane predicted localization; (ii) essentiality for both *T*. *gondii* and *Plasmodium* growth; and (iii) presence of predicted transmembrane domains. Taking a comparable approach, a recent review by Martin proposed novel, highly divergent candidate transporters for Pan in *P*. *falciparum* [10].

### The CoA biosynthesis pathway is conserved and essential in Apicomplexa

Pan is the precursor for the synthesis of CoA, which is an essential cofactor for a broad range of functions within all cells. CoA provides activated acyl groups and the prosthetic 4′-phosphopantetheine group for gene regulation, posttranslational modification of proteins via acetylation, and various metabolic functions, such as the tricarboxylic acid cycle (TCA cycle) and fatty acid synthesis (FAS) [48]. While differences are present in Archaea, the canonical pathway for CoA synthesis is present in most bacteria and eukaryotes [49]. The first step is the phosphorylation of Pan to 4′-phosphopantothenate, catalyzed by Pan kinase (PanK). This reaction is followed by the sequential formation of 4′-phosphopantothenoylcysteine by phosphopantothenoylcysteine synthetase (PPCS), 4′-phosphopantetheine by phosphopantothenoylcysteine decarboxylase (PPCDC), dephospho-CoA by phosphopantetheine adenylyltransferase (PPAT), and, finally, CoA by dephospho-CoA kinase (DPCK). Although this essential CoA pathway is highly conserved, there are differences between phyla and even between apicomplexan parasites (Fig 2) [20–22,36,50,51]. Most bacteria and *Saccharomyces cerevisiae* contain a single *PanK* [52]. Conversely, mammals harbor 3 *PanK* genes and 1 pseudo*-PanK* [53], which are expressed in different compartments and tissues [54,55] and have different regulatory properties [56]. The apicomplexans possess 2 distinct *PanK* genes, which have been thoroughly studied in *Plasmodium* species for their potential druggability. In *P*. *falciparum*, PanK1 and PanK2 are part of a macromolecular complex as heterodimers, a feature that is also conserved in *T*. *gondii* and unique to the Apicomplexa phylum [16,57]. *Pf*PanK1 is essential and the mutant *Pf*PanK2 generated by transposon insertional mutagenesis is predicted to have a reduced fitness [22,35]. Both *PanK*s are essential in *T*. *gondii* [16,57]. In these species, the heterodimerization is likely essential for the phosphorylation of Pan [57], whereas in *Plasmodium yoelii*, both *PanK*s are individually dispensable [51], indicative of a possible redundant function. Contrastingly and unexpectedly, the simultaneous knockout of both *PanK1* and *PanK2* appears to be viable in blood-stage *P*. *berghei* parasites [58]. Other unexpected cases that need further elucidation are the presence of 2 *PPCS* genes in *P*. *falciparum*, which are predicted to be dispensable, and the dispensability of *PfPPAT* [22]. For the latter, a nicotinamide nucleotidyltransferase (PF3D7_1327600) has been suggested as an alternative PPAT-encoding gene with an adenylyltransferase activity comparable to that of PPAT [59,60], possibly explaining the dispensability of PPAT. Studies in rodent malaria parasites have highlighted that single-copy *PPCS* and *PPCDC* are also dispensable [21,50]. The ability of PanK to phosphorylate pantetheine [61] points toward a possible salvage of pantetheine for CoA synthesis (Fig 1), similar to observations in some bacteria [62], potentially rendering *PPCS* and *PPCDC* dispensable. Nevertheless, the presence of pantetheine-hydrolyzing enzymes, pantetheinases (vanins), in the serum possibly limits the amount of freely available pantetheine. At this stage, the mode of a possible pantetheine uptake, much like that of Pan, remains elusive. This may occur through a conserved Pan transporter, or through diffusion, as has been suggested for 4′-phosphopantetheine [63]. Salvage of 4′-phosphopantetheine would make PanK activity also dispensable, possibly explaining the viability of the *PanK* double knockout in *P*. *berghei*. Interestingly, the presence of a single transporter for ketopantoate, pantoate, and CoA intermediates has recently been proposed in *Salmonella enterica* [64]; however, no homolog could be identified in Apicomplexa. While all these CoA synthesis enzymes, including *Tg*DPCK, are likely cytoplasmic, the last step performed by *Pf*DPCK has recently been localized to the apicoplast. This enzyme is essential for intra-erythrocytic parasite survival and remains active in the vesicles that accumulate after apicoplast disruption [36].

### Targeting the pantothenate and CoA synthesis pathways for drug development

**Fig 3:**
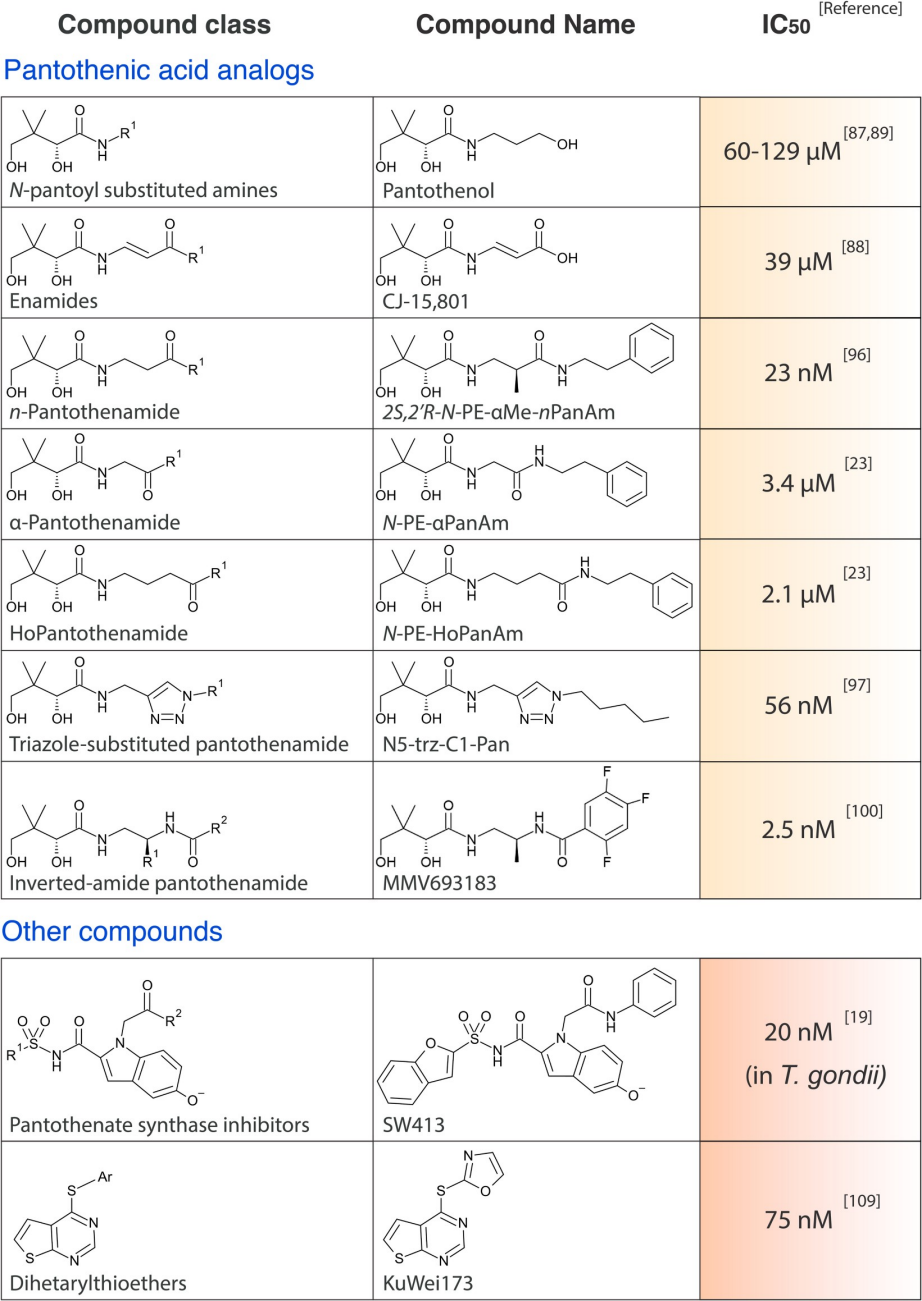
Pan and CoA synthesis targeting compounds. All IC_50_s are determined in *P*. *falciparum*, unless stated otherwise. CoA, coenzyme A; Pan, pantothenate.

While mammalian cells lack the ability to synthesize Pan, bradyzoites rely on this ability, making this pathway a plausible target for intervention against the chronic stages of *T*. *gondii* [16,65]. Interestingly, one of the proposed mechanisms of action of the current standard drug against *M*. *tuberculosis*, pyrazinamide, is the inhibition of Pan synthesis through the degradation of aspartate decarboxylase [66–69]. Unfortunately, the lack of an obvious aspartate decarboxylase homolog in *T*. *gondii* renders repurposing pyrazinamide for treatment of chronic *T*. *gondii* infections unlikely. Other targets for the inhibition of Pan synthesis in *T*. *gondii* may be KPHMT-KPR or PBAL [70]. While no inhibitors of the first enzyme have been identified, a panel of 13 Pan synthetase inhibitors that were designed against *M*. *tuberculosis* inhibited *T*. *gondii* growth with varying potencies, including SW413 (IC_50_ of 20 nM) (Fig 3) [19]. As discussed above, *T*. *gondii* tachyzoites do not rely on Pan synthesis *in vitro* [20], indicating that the activity of the Pan synthetase inhibitors may rather be a consequence of off-target effects. As parasiticidal activity of these compounds could be rescued by addition of excess Pan in the culture media, the off-target effect is potentially related to Pan uptake or CoA synthesis. Nevertheless, specific PBAL inhibitors may be efficacious against bradyzoites, though it should be noted that the limited permeability of the blood brain barrier and the cyst wall present an additional challenge to the design of drugs targeted to kill this stage of the parasite. *Plasmodium* species cannot synthesize Pan and therefore rely entirely on Pan uptake. The presumed divergence of the human and *Plasmodium* Pan transporter could be exploited to develop parasite-specific inhibitors, although this would require the identification of the parasite transporter in the first place [39,71].

Even though there are differences in the CoA biosynthesis pathway between apicomplexans, the majority of enzymes involved are essential for parasite survival (Fig 2), indicating their potential as antiparasitic drug targets. However, antimicrobials that target the Pan-fueled CoA biosynthesis pathway could have deleterious consequences in the host if its activity is not specific for the metabolism of the parasite. Defects in the CoA biosynthesis pathway in humans caused by mutations in *PanK*, *PPCS*, or *CoA synthase* (CoASY, a bifunctional PPAT/DPCK enzyme) lead to a variety of diseases including neurodegeneration and dilated cardiomyopathy [72–75]. In addition, mice fed on a Pan-free diet and treated with hopantenate, a Pan analog that inhibits PPCS [76], died within 5 to 15 days, even though no toxicity had been observed when concentrations of up to 800 μM were tested on a variety of cell lines [77]. The effects of hopantenate highlight the importance of thorough safety testing in animal models and developing compounds that are very specific and ideally target parasite enzymes only.

The dispensability of *PPCS* in *Plasmodium* and *PPCDC* in rodent malaria parasites precludes these 2 enzymes as ideal drug targets [21,22,50]. In contrast, the bifunctional CoaBC enzyme (with both PPCS and PPCDC function) in *M*. *tuberculosis* is a potential drug target based on the bactericidal effects upon knockdown [70,78]. More promising targets in *P*. *falciparum* are PanK1, DPCK, and, possibly, PPAT. This is especially the case for *Pf*PPAT, if essential, which exhibits weak homology to the human bifunctional PPAT/DPCK enzyme CoASY [79]. PanK1 and DPCK have higher homology to the human equivalents, and this would require the development of parasite-specific compounds. The sequence homologies of the CoA synthesis enzymes and the potential to develop *Plasmodium*-specific compounds is extensively reviewed in Spry and colleagues’ paper [80].

Already since the 1940s, the CoA synthesis pathway has been considered and explored as a drug target [81]. Following the discovery of the structure of Pan [82], many Pan analogs have been generated that all act as antimicrobials [81]. Interestingly, some of these also showed weak activity against avian, nonhuman primate and human malaria parasites [83–85]. In 2005, 2 Pan analogs, pantothenol (provitamin B5) and CJ-15,801 (a natural fungal product) [86], were shown to have micromolar activity against *P*. *falciparum* asexual blood stages (Fig 3) [87–89]. More promising results were obtained with pantothenamides (PanAms), a class of Pan analogs that first showed micromolar activity, but potency was improved to the low nanomolar range by inhibiting or inactivating the pantetheinase enzymes present in the human serum [23,90–92]. These enzymes hydrolyze pantetheine to Pan and cysteamine and can also degrade PanAms (Fig 1) [91,93,94]. Different strategies have been used to generate PanAms that are resistant to degradation, including (i) addition of a methyl group adjacent to the distal amide [95,96]; (ii/iii) displacement of the distal amide bond in the pantothenamide structure by removal or addition of a methylene group in its β-alanine moiety producing α-PanAms and HoPanAms, respectively [23]; (iv) replacement of the pantetheinase-susceptible amide bond by a triazole isostere to make the triazole-substituted PanAms [97,98]; and (v) inversion of the labile amide bond, leading to the inverted-amide PanAms (iPanAms) [99,100]. This led to very promising drug candidates, including *N*-PE-αMe-*n*PanAm [96], N5-trz-C1-Pan [97], and MMV693183 with IC_50_s of 23 nM, 56 nM, and 2.5 nM, respectively, against asexual blood-stage *P*. *falciparum* (Fig 3). Although these strategies also led to stable and active compounds against gram-positive bacteria, they only showed micromolar activity in these organisms [101–103]. The lack of activity of an N7-PanAm and, possibly, other derivatives, against the gram-negative bacteria, *E*. *coli*, is partially due to the efflux of the drug through TolC-dependent pumps [104]. PanAms have also shown potent activity against *Plasmodium knowlesi* [105], *Plasmodium vivax* [100], *P*. *falciparum* sexual blood stages *in vitro*, and asexual blood stages in an *in vivo* humanized mouse model [99,100]. In addition, the iPanAm MMV688558 inhibits *T*. *gondii* growth *in vitro* with an IC_50_ of 0.95 μM [106], showing its potential to target other apicomplexan parasites.

The potent antimalarial activities of PanAms show that the Pan and CoA synthesis pathways are indeed plausible drug targets. Drug screenings have been performed to find compounds that are chemically different to Pan analogs and could target CoA synthesis. Recently, a library of compounds was tested to inhibit *Pf*PanK1 activity and *P*. *falciparum* growth, and 4 inhibitors were identified with micromolar activity that may serve as new scaffolds [61]. Furthermore, a chemically diverse set of inhibitors against *P*. *falciparum* was suggested to target the CoA synthesis pathway, based on their reduced activity upon supplementation with metabolites from the CoA pathway [107,108]. One of the putative CoA biosynthesis targeting drugs, Amb180780, is part of the dihetarylthioether class of drugs that has been further developed into the promising new candidate, KuWei173 (Fig 3) [109]. A structurally related compound, compound 33 from Edlin and colleagues, reduced parasitemia by 34% in a rodent malaria model [110]. Activity of Amb180780 on *Trypanosoma brucei brucei* [107] and the potent dihetarylthioethers make these compounds an exciting basis for new drugs that may target the CoA pathway in diverse protozoan parasites belonging to the kinetoplastids and apicomplexans.

### Mechanism of action of pantothenate analogs

The mechanism of action of the Pan analogs has been a major topic of investigation and debate for the last 2 decades, with an obvious focus on the CoA biosynthesis pathway. It was first noticed that Pan analogs compete with Pan, leading to a reduction in the formation of phosphorylated Pan (4′-P-Pan) [87–89,97,99]. However, this was not due to competition with the uptake of Pan, since accumulation of intracellular PanAm does not correlate with its antiplasmodial activity. Furthermore, inhibition of the Pan transporter by phloretin blocked uptake of α-PanAms, but increased the uptake of HoPanAms, *N*-PanAms, and pantothenol [39,111,112]. The formation of phosphorylated PanAms (4′-P-PanAm), and the identification of CJ-15,801- and pantothenol-induced mutations in *PanK*, suggested that this kinase may be targeted [35]. However, the antiplasmodial activity of PanAms does not correlate with the inhibition of Pan phosphorylation [97,112] or PanK activity [61,99]. Using a PanAm that is capable of binding PanK without being metabolized, de Villiers and colleagues convincingly demonstrated that PanK is not the target but rather the metabolic activator of these compounds [112].

Whether PanAms are effective against a certain organism depends on the type of PanK these organisms are expressing, type I (PanK_I_), type II (PanK_II_), or type III (PanK_III_). Bacteria that express PanK_I_, e.g., *E*. *coli*, are sensitive to PanAm treatment, while bacteria with PanK_III_, e.g., *Pseudomonas aeruginosa*, are resistant. This is due to the high specificity of PanK_III_ for Pan and therefore excludes binding of PanAms or pantetheine. In contrast, PanK_I_ can accept PanAms and pantetheine as substrates and convert them into P-PanAms or P-pantetheine, respectively [102,113–115]. *Staphylococcus aureus* and eukaryotes, including *Plasmodium*, express PanK_II_ and are also able to phosphorylate PanAm [35,99,102,116].

After activation by PanK, phosphorylated Pan analogs are further metabolized to varying degrees and converted into antimetabolites by the enzymes of the CoA pathway. In bacteria, CJ-15,801 and pantothenol are metabolized to form a substrate for PPCS that blocks this enzyme’s activity in a competitive manner, which has also recently been shown for hopantenate in humans and *Drosophila* [76,117,118]. In contrast, PanAms are converted into the corresponding CoA antimetabolites (CoA-PanAms) that inhibit CoA-utilizing enzymes in bacteria, like the acyl carrier protein (ACP) involved in FAS [104,119,120]. While *S*. *aureus* metabolizes PanAms into the antimetabolites that could inhibit CoA-utilizing enzymes [119,121], these bacteria release the phosphorylated product very slowly [116]. It is therefore suggested that P-PanAm, which remains bound to PanK, is the major determinant of inhibiting PanK_II_ by PanAms in *S*. *aureus* instead of inhibiting CoA-utilizing enzymes [102,116,122]. Interestingly, CoA-PanAm can bind to the regulator of aspartate decarboxylase, PanZ, in *E*. *coli* and thereby inhibit the formation of β-alanine, which is required for Pan synthesis [123–126]. This is an additional mechanism of action of PanAms along with inhibiting CoA-utilizing enzymes. However, PanZ is conserved in γ-proteobacteria only, and, therefore, this mechanism of action does not apply to other bacteria, like *M*. *tuberculosis* [123]. If *T*. *gondii* relies on β-alanine synthesis, it would be interesting to further investigate whether such a feedback loop exists and explore this pathway for compound development. The difference in the mechanism of action of Pan analogs between bacteria highlights that these compounds may also exhibit distinct mechanisms of action in *Plasmodium*.

**Fig 4:**
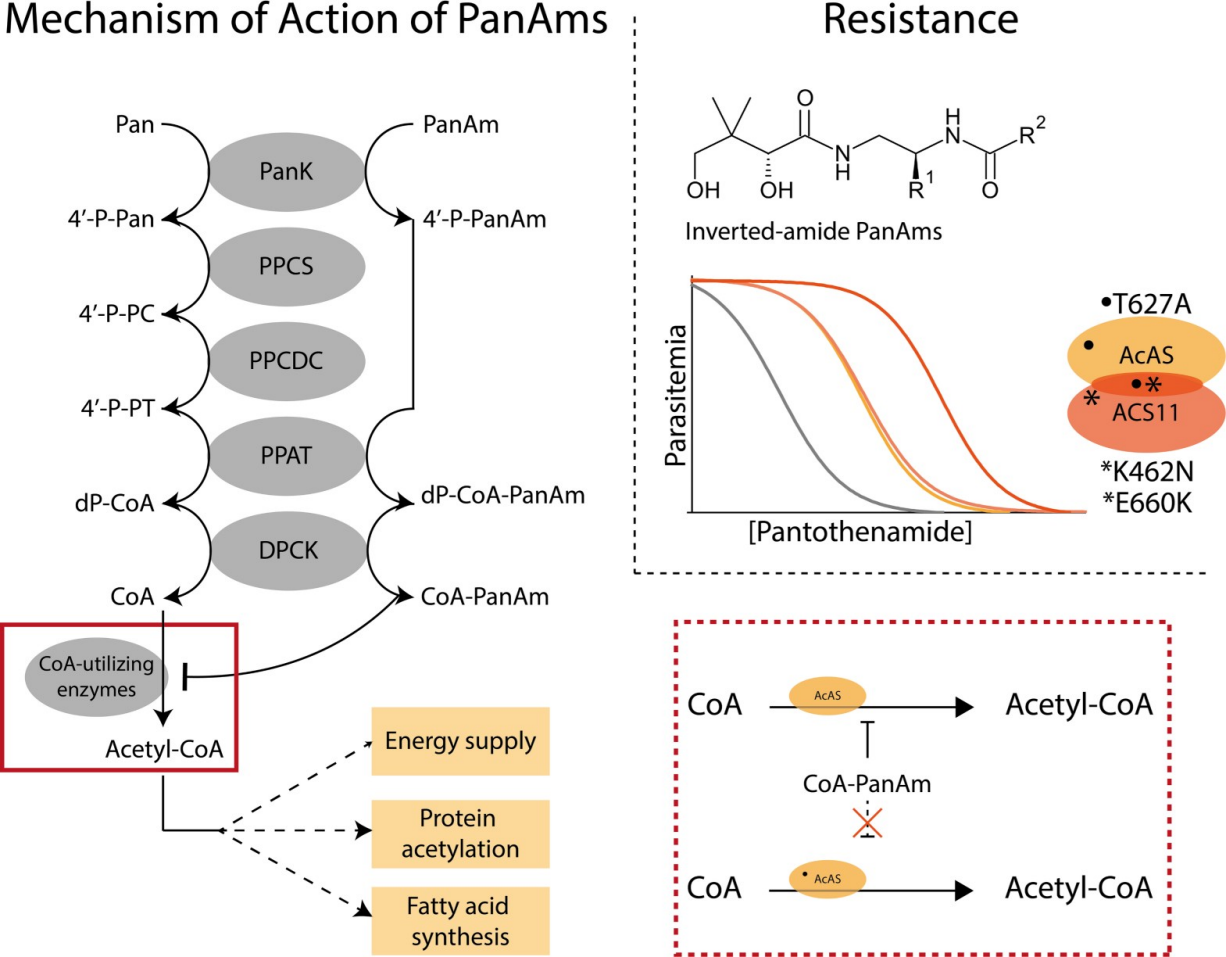
Mechanism of action of PanAms. PanAms are converted into CoA antimetabolites (CoA-PanAms) using 3 enzymes of the CoA pathway and reduce acetyl-CoA levels. Mutations in AcAS and ACS11 determine the resistance phenotype against iPanAms, which is indicated by the increased concentration of PanAms needed to kill parasites with mutations (yellow and orange lines) compared to wild-type parasites (gray line). Parasites are more resistant when both AcAS and ACS11 are mutated (dark orange line) than parasites with a single mutation in one of these enzymes (yellow and light orange lines). CoA-PanAm blocks the activity of the CoA-utilizing enzyme AcAS (red boxes), thereby reducing acetyl-CoA levels that may lead to downstream effects on protein modification or fatty acid metabolism in asexual blood stages. It is hypothesized that CoA-PanAm cannot bind to the mutated AcAS (dashed line), resulting in a normal level of acetyl-CoA. Whether ACS11 is a target of CoA-PanAm is still unknown. AcAS, acetyl-CoA synthetase; ACS11, acyl-CoA synthetase 11; CoA, coenzyme A; CoA-PanAm, pantothenamide CoA-analog; DPCK, dephospho-CoA kinase; dP-CoA, dephospho-CoA; dPCoA-PanAm, pantothenamide dephospho-CoA-analog; iPanAms, inverted-amide PanAms; Pan, pantothenate; PanAm, pantothenamide; PanK, pantothenate kinase; PPAT, phosphopantetheine adenylyltransferase; PPCDC, phosphopantothenoylcysteine decarboxylase; PPCS, phosphopantothenoylcysteine synthetase; 4′-P-Pan, 4′-phosphopantothenate; 4′P-PanAm, 4′-phosphopantothenamide; 4′-P-PC, 4′-phosphopantothenoyl-L-cysteine; 4′P-PT, pantetheine-4′-phosphate.

In *P*. *falciparum* parasites treated with the iPanAm MMV689258, acetyl-CoA levels were reduced while CoA levels remained stable. Furthermore, induction of resistance to iPanAms led to mutations in the acetyl-CoA synthetase (AcAS [127]) and acyl-CoA synthetase 11 (ACS11). Confirmation of the role of these mutations in drug sensitivity using CRISPR-Cas9 gene editing in combination with extensive metabolomic profiling demonstrated that PanAms are also converted into CoA-PanAms, and, recently, it has been conclusively shown that these CoA-PanAms inhibit AcAS activity (Fig 4) [35,99,100]. Whether PanAms target other CoA-utilizing processes, such as is the case in bacteria, is still unknown [104,119,120]. Contrastingly, the mechanisms of action of CJ-15,801, pantothenol, and other Pan analogs in *P*. *falciparum* have not been studied further. Based on the dispensability of *PPCS* in *Plasmodium* asexual blood stages, the possible redundancy of the enzyme, and the putative existence of an alternative pathway for CoA production, PPCS may not be the target [21,22,50,62,63]. However, it remains to be further explored whether these Pan analogs could target both PPCS enzymes and/or other downstream enzymes.

### Downstream effects of pantothenamides in Apicomplexa

The findings reported to date indicate that the mechanism of action of PanAms in *Plasmodium* is based on their conversion to CoA-PanAms that inhibit CoA-utilizing processes and the generation of acetyl-CoA [35,99,100]. However, the exact downstream effects remain unknown. Although AcAS was identified as a target, there are 3 common enzymes and complexes in *Toxoplasma* and *Plasmodium* parasites that are able to produce acetyl-CoA and may therefore be targeted by CoA-PanAms: (i) the mitochondrial branched chain ketoacid dehydrogenase (BCKDH) complex [17,34,128]; (ii) the pyruvate dehydrogenase (PDH) complex in the apicoplast [129,130]; and (iii) the cytosolic and nuclear AcAS [100,127,131]. *T*. *gondii* parasites possess a fourth enzyme that can produce acetyl-CoA, the ATP-citrate lyase (ACL) [132–134]. Acetyl-CoA generated by the BCKDH complex is funneled into the TCA cycle; however, asexual blood-stage malaria parasites largely rely on glycolysis for ATP production, and a complete TCA cycle becomes essential only during transmission [34,135–138]. While *T*. *gondii* parasites produce most ATP through oxidative phosphorylation, BCKDH is not essential and the parasites readily switch to utilizing a partial TCA cycle fueled by glutamine in its absence [34,139]. The PDH complex provides acetyl-CoA for the FASII pathway within the apicoplast. Although FAS is inhibited by CoA-PanAms targeting ACP in *E*. *coli* [104,119], PDH and the FASII pathway are dispensable during *Plasmodium* blood-stage development and only become important in mosquito and liver stages [128,140,141]. Similarly, loss of PDH and FASII subunits are only associated with a substantial fitness defect but fully rescuable through provision of excess exogenous fatty acids (FAs) in *T*. *gondii* tachyzoites [142,143]. It is currently still unknown whether CoA-PanAms can inhibit these enzymes. Based on their dispensability in asexual blood stages, it is unlikely that inhibition of BCKDH and/or PDH account for the potent activity of PanAms in these stages.

**Fig 5:**
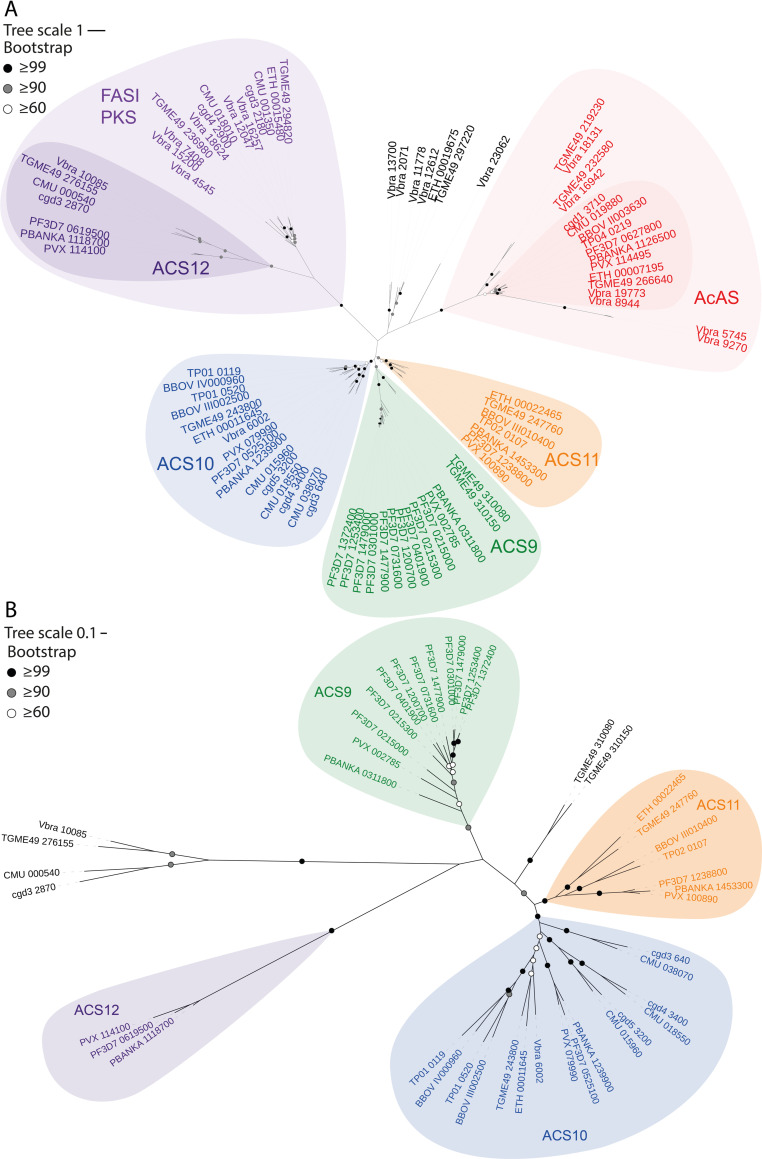
Phylogenetic tree of ACS and AcAS. Phylogeny of the ACS family and AcAS in Apicomplexa. The hits identified with the iterative jackhmmer search (S1 Table; [160]) were used for the phylogenetic analysis. *Pf*ACS1b was not identified based on its homology to the query sequences in the jackhmmr search but was added based on previous literature [154]. MegaX was used to align all hits with ClustalW, followed by building an unrooted tree using a maximum likelihood analysis with a bootstrap phylogeny test. In initial analyses, 5 hits were excluded because these sequences did not cluster with any of the annotated family members. (A) Phylogenetic tree of all identified ACS and AcAS family members. Different settings were tested, including the alignment of the full amino acid sequence or the AMP-binding enzyme (PF00501) domain sequence, followed by a maximum likelihood analysis using all sites or a 50% partial deletion, all leading to comparable results. Phylogenetic tree analyses with 50% partial deletion excluded 1 hit because this alignment aligned with less than 50% of the genes. The final phylogenetic tree presented here is based on the full amino acid sequence of the identified ACS and AcAS family members, analyzed by the maximum likelihood method, 50% partial deletion with a bootstrap phylogeny test with 500 replicates. (B) Phylogenetic tree of only ACS family members. The phylogeny of the clusters of ACS family members was further tested in a maximum likelihood analysis using all different approaches as under (A). In none of the analyses, TGME49_310080 and TGME49_310150 clustered with ACS9, while the *Plasmodium* ACS12 cluster only once included the additional sequences from (A). The final phylogenetic tree presented here is based on the full amino acid sequence, analyzed by the maximum likelihood method, 50% partial deletion with a bootstrap phylogeny test with 100 replicates. AcAS, acetyl-CoA synthetase; ACS, acyl-CoA synthetase; FASI, type I fatty acid synthase; PKS, polyketide synthase.

In contrast, the third enzyme that produces acetyl-CoA, AcAS, has been convincingly demonstrated to be targeted by CoA-PanAm [100] and other inhibitors [127] and is essential in *Plasmodium* asexual blood stages [22,127]. The essentiality of AcAS and its potent inhibition through PanAms likely explain the deleterious effect of PanAm treatment on *Plasmodium*. In contrast, genetic disruption of *TgAcAS* and *TgACL* shows that these genes are individually dispensable for *T*. *gondii* parasites survival, while disruption of both genes is lethal, indicative of an overlapping function [132–134]. Both enzymes have been shown to be important for FA elongation (FAE) and N-ε-lysine acetylation of histones and non-histone proteins [133]. *Pf*AcAS also provides acetyl-CoA for histone acetylation and has been associated with a chromatin-remodeling complex [127,131]. The FAE pathway is essential for both *T*. *gondii* and *P*. *falciparum* parasites since it can generate very long chain FAs that cannot be provided by the host cell [133,134,144,145]. In addition, N-ε-lysine acetylation of non-histone proteins and histones is widespread in both parasites [146,147]. The latter plays a major role in parasite development and viability [148–152]. In contrast, the role of acetylation of non-histone proteins, although widespread, is not well understood [133] but has also been shown to be essential in some cases, such as for α-tubulin in *T*. *gondii* [153]. AcAS is well conserved across the apicomplexans (Fig 5), and, therefore, it could be hypothesized that *Plasmodium* AcAS may also be involved in FAE pathway and N-ε-lysine acetylation, in addition to histone acetylation. PanAm treatment, targeting AcAS, may therefore alter FA elongation, gene regulation, and protein function, leading to parasite death (Fig 4). As these processes are predicted to occur in the cytosol and nucleus, this would explain why BCKDH and PDH cannot rescue the phenotype after PanAm treatment.

The second mutation identified in iPanAm-resistant parasites was on conserved amino acids in the CoA binding site of *Pf*ACS11 [99], which is an acyl-CoA synthetase. The function of this enzyme in *Plasmodium* parasites, and whether ACS11 is a PanAm target or otherwise contributing to resistance is still unknown. Phylogenetic analysis shows that the 13 acyl-CoA synthetases found in *P*. *falciparum* [154] cluster into 4 separate subgroups grouping with ACS9, ACS10, ACS11, and ACS12 (Fig 5A and 5B). Members of the ACS10 and ACS11 subgroups, identified to be most similar in *P*. *falciparum* (Fig 5B) [154], are common across the Apicomplexa phylum, although acyl-CoA synthetases from *Cryptosporidium* spp. exclusively clustered with ACS10. Seven homologs are found in *T*. *gondii*. Two candidates clustered with ACS10 and ACS11, two with ACS9, and one with ACS12 that included a *Cryptosporidium* homolog, although these latter two could not be confirmed by further analyses (Fig 5B). One did not cluster with any subgroup, and the propionate-CoA ligase clustered with the AcASs subgroup, suggesting a short chain acyl-CoA synthetase function. It has been suggested that acyl-CoA synthetases are involved in FAs scavenging from the host [155]. Individual acyl-CoA synthetases from *Cryptosporidium parvum* and *P*. *falciparum* have different localizations and expression patterns [156,157], and the number and distinct types of acyl-CoA synthetases vary among the apicomplexans (Fig 5) [154,155]. Taken together, this suggests that acyl-CoA synthetases play different roles in FAs scavenging throughout the life cycle, possibly depending on nutrient availability in different host/host-cell niches and on stage-specific parasite metabolism. Two chemically distinct compounds, Triacsin C, which is a polyunsaturated FA, and PanAms, are able to inhibit *C*. *parvum* and *P*. *falciparum* growth, respectively. Triacsin C inhibits *Cp*ACS1 (cgd3_640) and *Cp*ACS2 (cgd5_3200) activity [158], while CoA-PanAms may inhibit *Pf*ACS11 [99]. However, the potential of *Pf*ACS11 as a drug target is still unknown as it is unclear whether ACS11 plays an essential role [22,127]. Additionally, the induction of mutations in *ACS11* by exposure to chemically unrelated compounds [127,159] suggests that this enzyme may be a general marker of resistance and not the specific target of PanAms. While *Cp*ACS1 and *Cp*ACS2 cluster with the ACS10 subgroup, which is most similar to the ACS11 subgroup (Fig 5), it would be interesting to investigate if ACS10 and ACS11 are potential drug targets of FA analogs in other apicomplexans.

## Conclusions

It is well known that Pan is an essential nutrient to produce CoA via a highly conserved pathway, but with striking differences between apicomplexan parasites. Understanding the processes of Pan uptake, synthesis, and metabolism into CoA in all apicomplexans is crucial for drug development. Recently, new insights into CoA metabolism and a potential new drug target, the Pan synthesis pathway, for *T*. *gondii* bradyzoites have been revealed. However, the dispensability or essentiality of some enzymes of the CoA synthesis pathway remains unclear and needs to be addressed first. This limited understanding notwithstanding, CoA metabolism is demonstrated to be an excellent target. One prime example is the potent activity of PanAms against CoA-utilizing enzymes in *P*. *falciparum*. The mode of action through AcAS may lead to downstream effects on protein acetylation and FA metabolism. Overall, the CoA requirements for FA elongation, protein acetylation, and other processes in apicomplexan parasites indicate the potential of PanAm or other Pan and CoA synthesis-targeting compounds to act as broad anti-parasitic drugs.

## Supporting information

S1 Table Orthologs of acyl-CoA synthetases and AcAS identified for phylogenetic analysis. The full amino acid sequences of *Pf*ACS9, *Pf*ACS10, *Pf*ACS11, and *Pf*AcAS, and the partial amino acid sequence of *Pf*ACS12 (the first 664 amino acids, excluding the thiamin-diphosphate-binding fold domain) were used as query sequence (green) in an iterative search (jackhmmer) (5 iterations) for orthologs in the UniProtKB database [160]. E-values found in our jackhmmr search are reported, and hits were identified as genes that showed an E-value of <10^−50^ for at least 1 query sequence. The E-value of genes that were below the E-value cutoff for a query sequence are indicated in red. The following genes were excluded from phylogenetic analysis: genes that were not identified as hits as the E-value was below the cutoff for all query sequences (gray shading), genes that did not align using the partial deletion cutoff of 50% in our alignment using ClustalW (dark gray shading), and genes that did not cluster with other genes in the phylogenetic tree (blue shading).
(XLSX)
